# Explaining the Role of Personal, Social and Physical Environment Factors on Employed Women's Physical Activity: A Structural Equation Analysis

**DOI:** 10.5539/gjhs.v5n4p189

**Published:** 2013-05-13

**Authors:** Fatemeh Bakhtari Aghdam, Mohammad Hossein Baghiani Moghaddam, Mohammad Asghari Jafarabadi, Hamid Allahverdipour, Saeed Dabagh Nikookheslat, Roghaiyeh Nourizadeh

**Affiliations:** 1 Department of Public Health, Shahid Sadoughi University of Medical Sciences, Yazd, Iran; 2 Medical Education Research Center, Faculty of Health, Tabriz University of Medical Sciences, Tabriz, Iran; 3 Clinical Psychiatry Research Center, Department of Health Education & Promotion, Tabriz University of Medical Sciences, Tabriz, Iran; 4 Department of Sport Physiology, Tabriz University, Tabriz, Iran; 5 Department of Nursing & midwifery, Shahid Beheshti University of Medical Sciences, Tehran, Iran

**Keywords:** physical activity, individual factors, environmental factors, Iranian women

## Abstract

**Background and Objectives:**

PA is a multi-factorial behavior that is affected by interpersonal, intra personal, environmental and social factors. In this study we applied explanatory model to determine the total, indirect and direct impact of physical environment, personal factors and social support on PA among employed women.

**Methods:**

This study was a correlational cross-sectional study which was conducted to model total, indirect and direct impact of environmental, psychological and social factors on PA. A total of 200 women were chosen from Tabriz University by using convenience sampling method. Data about demographic characteristics, psychological variables, social and physical environment were gathered by using self-reported questionnaire and also the PA was measured by using the International PA Questionnaire and pedometer.

**Results:**

personal factors, physical and social environment, showed direct effects on PA. Social factors could be seen to have indirect effects on PA through their influence on personal factors such as pros, cons and self-efficacy; also physical environment had indirect effects on PA through social environment. The total effects of physical and social environment on PA type were respectively 0.17, 0.16 on walking, 0.05, 0.07 on moderate activity and 0.15, 0.18 on vigorous activity.

**Conclusions:**

Findings from this study indicated that social factors had indirect effects on walking, moderate and vigorous activity, especially through the effects on these factors of self-efficacy, physical environment, pros and cons, and the interactive role of individual, environmental and social impacts on PA. The current study identifies that psychological, physical and social factors could be shown to have direct and indirect influences on all forms of activity. The barriers of PA were the most predictor of this behavior, and based on results, it can be concluded that decreasing the barriers along with improving social and physical environment can lead to increasing PA and health promotion.

## 1. Introduction

There are comprehensive evidences in the literature that indicate PA is an inseparable part of healthy lifestyle of individuals and communities which has numerous healthy outcomes (Faghri et al., 2008; Huang et al., 2010; Dunn & Blair, 2002; Shibata et al., 2009) including the prevention of cardiovascular diseases, diabetes and cancers, hypertension, osteoporosis and other chronic diseases. PA is also stated to be crucial for rehabilitation, prevention of obesity (Aittasalo et al., 2004; Cerin et al., 2009; Bolívar et al., 2010). Additionally PA is known to be as one of the important behaviors to improve healthy lifestyle which leads to body fitness, self-satisfaction, promoting social interaction, mental performance, and physical function (Latham et al., 2003; Taylor et al., 2004). PA, as a multidimensional behavior, is affected by numerous interpersonal, intra personal, environmental and social aspects (Khan et al., 2002; McNeilla et al., 2006; Pan et al., 2009; Heath et al., 2010; Huang et al., 2010; Ishii et al., 2010). It has been shown and emphasized that physical and social environment influence the ability of people to perform PA (VCE, 2010). In addition, the recent evidences have indicated that the relationship between environmental-social factors and physical activities (Khan et al., 2002; Donnelly et al., 2009; Shibata et al., 2009; Bolívar et al., 2010; Huang et al., 2010), is consistent and in the line of ecological models in which behaviors are affected by interaction between personal, environmental and psychosocial factors (Shibata et al., 2009; Ishii et al., 2010). Furthermore, there is a large number of studies about the interaction and relationship of effective factors that explain PA related to behaviors which indicate necessity of auditing and clearing how these personal-social–ecological factors predict PA in relation to behaviors. However, many of the explanatory models focus on personal factors such as self-efficacy rather than other factors such as social and physical environment. There are some studies that address ecological factors (Humpel et al., 2002). There is a growing tendency to apply ecological models as a more productive framework for promoting PA (Sallis et al., 2006; Elder et al., 2007; Shibata et al., 2009; Ishii et al., 2010). In the field of public health, ecological models describe people's interactions with their physical and socio-cultural surroundings (Elder et al., 2007; Shibata et al., 2009; VCE, 2010). Ecological models are distinguished by their definite inclusion of environmental and policy variables that are expected to affect behavior. Ecological models incorporate a wide range of influences at multiple levels of variables rather than positing behavior under the influence of personal variables (Sallis et al., 2006; Elder et al., 2007; Ishii et al., 2010). Ecological models of PA often include intrapersonal, social environment, physical environment, and policy (Sallis et al., 2006). Findings from studies (Sallis et al., 2006; Elder et al., 2007; Cerin et al., 2009; Cleland et al., 2010; Huang et al., 2010; Ishii et al., 2010) that used socio ecological model displayed that these models are useful in attempting to understand the influences on PA behaviors. Also it has been indicated that social support may create social interaction opportunities that lead to better sense of community. High levels of social support can facilitate PA in the physical environment and lead to increase self-efficacy (McNeill et al., 2006; McNeilla et al., 2006; Elder et al., 2007; Ishii et al., 2010). The study of McNeill et al. (2006) showed that both social and physical environmental factors influenced PA indirectly through personal factors, which in turn influenced PA; in addition, physical environment was found to have a direct effect on PA. This study indicated that both social and physical environmental factors and personal attributes were associated with walking, moderate-intensity activity and vigorous activity. Another study suggested that environmental factors had indirect effects on walking, moderate and vigorous activity through social support and individual factors; likewise social support influenced PA through self-efficacy indirectly (Ishii et al., 2010).

Findings indicate that women, especially in the developing countries were known to be at the greater risk of inactivity (Elder et al., 2007; Bolívar et al., 2010; Cleland et al., 2010; Ramadan et al., 2010), and previous findings have shown that women have less mobility than men (Aittasalo et al., 2004; Heath et al., 2010). In addition, in Islamic countries because of cultural and religious beliefs and norms women are not obliged to perform physical activities. In addition, the nature of clerical jobs and using computer and internet at workplaces could decrease employees’ physical activities (Sallis et al., 1998; Huang et al., 2010). Employed women's physical activities and influencing factors is mostly investigated and reported in different cultures and populations (Cervello et al., 2010). Numerous findings in Iran showed that nearly 75% of Iranian women were inactive at leisure time (WHO., 2005). Because of cultural and environmental context of women as a Muslim community, explaining the determinant factors on PA would help to facilitate PA promotion programs. Beside no study, if any, examined PA based on pedometer in Iranian women. In accordance with the above-mentioned background, the purpose of the present study was to explain direct, indirect and total effects of personal, social and physical environmental influences on Iranian women's PA behaviors. Additionally, as countries have their own psychosocial and cultural dimensions, the current study focuses on responses of Iranian women to deliver PA promotion programs.

## 2. Methods

A total of 200 women (Bryant & Yarnold, 1995; DeVellis, 2003) were chosen from Tabriz University located in East Azerbaijan province at the North Western part of Iran using convenience sampling technique. Age (year), number of children, employment status, marital status and level of education were evaluated in the self-administered questionnaire. Participants reported the most suitable from the categories of employment status (official employees of the government, worked on contract-based situation, semi-official and private employment status), history of PA (yes, no), level of education (no high school diploma, high school graduate, associate degree, BSc, MSc and doctorate degree), marital status (currently married, currently single). All employees were informed by an internet message (email) of the study purposes (to evaluate PA through a questionnaire and pedometer registration). The researchers personally sought employees’ participation one week later. Those willing to participate were given information about the procedures, a questionnaire, a pedometer, an activity log and a guide on how to apply pedometer and the activity log. Researchers collected the questionnaires, activity logs and pedometer.

The level of PA was measured by the Iranian version of the long form of the International PA Questionnaire (IPAQ) to assess self-report PA. This self-administered questionnaire evaluated PA at work, during transportation, during domestic and gardening activities and during leisure time (L-T), time spent on sitting based on the guidelines for data processing and analysis of the IPAQ. Total scores for PA extracted in MET- minutes/week, were calculated. Furthermore, the total number of walking, moderate and vigorous PA was calculated according to the IPAQ protocol (2005).

The metabolic equivalent scores were converted to MET in the IPAQ, for each type of activity by multiplying the number of minutes performed to each activity class by the specific MET score for that activity. One MET is equal to 3.5 ml O_2_ kg^-1^min^-1^ and is resting metabolic rate during quite sitting (Hagstromer et al., 2008). Self-reported PA level was classified as ’low’ (MET ≥ 600), ’moderate active’ (600 < MET < 3000) and ’vigorous activity’ (MET > 3000).

The IPAQ is known as valid and reliable instrument to evaluate PA in the previous studies (Craig et al., 2003; Vasheghani-Farahani et al., 2011; Baghiani Moghaddam et al., 2012). Pedometers were used to step count. Pedometer- based PA level was categorized according to baseline step counts into 'sedentary – low active’ (0-7499 steps/day), ’moderately active’ (7500-9900 steps/day) and ’active’ (> 10000 steps/day) (puig-ribera, mckenna et al., 2008).

### 2.1 Psychological Variables

The exercise self-efficacy scale (ESES) developed by Bandura (1997) consisted of 18 items rated using a four-point likert scale ranging from 1 (strongly agree) to 4 (strongly disagree). The validity and reliability of this scale was 0.87, 0.69.

The measurement of exercise benefit/barrier scale (EBBS) developed by Sechrist et al. (1987) was utilized to estimate the perceived positive (Pros) and negative aspects. (Cons) The questionnaire consisted of 28 item pros scale and 14 item cons scale which is rated using a four point likert scale ranging from (1 strongly agree) to 4 (strongly disagree). The validity and reliability of these scales were 0.81, 0.66.

### 2.2 Social Variables

Social support was measured by summing responses to frequency in three past months friends/work colleagues engaged in PA with participants or encouraged them to be physically active (Cleland et al., 2010). This scale included 5 items that were rated using a five – point likert scale ranging from 1 (seldom) to 5 (always). The validity and reliability of this scale were 0.77, 0.72.

### 2.3 Environmental Variables

Employees’ perceptions of their neighborhood environment were assessed by an eight items measure including “My neighborhood offers many opportunities to be physically active”. “Local gyms and other facilities in my neighborhood offer many opportunities to get exercise”; “my neighborhood provides facilities to walk”, “It is pleasant to walk in my neighborhood”, “in my neighborhood it is easy to walk places”, “the trees in my neighborhood provide enough shade”, “I frequently observe other people exercising”, “My neighborhood provides a safe and well-maintained environment (e.g., adequate lighting and light traffic volume.” Each item was measured using a five-point likert scale. Ranging from 1 (strongly agree) to 5 (strongly disagree) (Cleland et al., 2010). The validity and reliability of this scale were 0.76, 0.74.

### 2.4 Statistical Analyses

LISREL version 8.52 (Jöreskog & Sörbom, 1993) was performed to test the fitting of PA models to data extracted from participants. Data were presented by mean (SD) and Frequency (Percent) for quantitative and qualitative variables respectively. Multiple imputation in EM algorithm method was run to manage missing data (Allison, 2003). To determine the relationship between physical environment, social support and psychological factors with PA path analysis was used as a tool of structural equation modeling (SEM) (Ishii et al., 2010). Researchers were enabled to exam a series of regression equations by SEM. It is proposed base on ecological model correlation between psychological factors, social support and physical environment with all forms of activity and examined direct, indirect and total effects among variables. In the theoretical model, it is assumed that social factors influence PA through personal factors. Physical environment influences PA through personal and social factors.

The current study indicated no significant association between thus this path was omitted.

We reported path coefficients and correlations as standardized estimates. Two primary tests were conducted to survey data fit. The practical indicators of fit, according to CFA, include Chi-Square, Root Mean Square Error of Approximation (RMSEA), Root Mean Square Residual (RMSR), Goodness-of-fit index (GFI), comparative fit index (CFI) and adjusted goodness-of-fit index (AGFI).

The values for GFI, AGFI and CFI range from 0 to 1 and are derived from comparing a hypothesized model with the independent model; with a value greater than 0.90 indicating an acceptable fit to the data. Conventionally, there will be a good model fit if RMSEA is less than/equal to 0.08 and RMSR is less than 0.05. There is adequate fit if the RMSEA is less than/equal to 0.08 and RMSR is less than 0.05 (Bentler & Bonett, 1980; Bentler, 1990; Kline, 2004). P-Values < 0.05 considered as statistically significant.

## 3. Results

The women reported about their education, 3.4 per cent did not have high school diploma, 17.2 per cent were high school graduates, 8.3 per cent had associate degree, 53.8 per cent with a BSc, 15.2 per cent with MSc and finally 2.1 per cent had a doctorate degree. The mean of age for these women was 36.8 years.

A percentage of 38.7 per cent were official employees of the government, 43.7 per cent worked on contract-based situation, 10.6 per cent had semi-formal and 7 per cent were of private employment status. Among them 22.3 per cent were single and the rest were married. As such, 35.9 per cent had no children, 33 per cent one, 28.3 per cent two, 1.4 per cent three and 1.4 per cent had four children.

According to the IPAQ protocol, the median was calculated for different domains of PA. The highest domain was domestic and yard PA. The results are displayed in Table 1.

**Table 1:** The median for different domains of PA

PA ( MET- min week-1)	Med (Q1-Q3)
PA at work	100 (0-350)
Transportation PA	198 (0-355.5)
Domestic and yard PA	460 (140-1080)
L-T PA	354.2 (103.1 1362)
Total PA	1770 (745-3718.5)

PA: physical activity, L-T: leisure time

Eighteen percent of participants reported low level of PA, 50 per cent reported moderate PA and 32 per cent reported vigorous PA. The mean ± SD of pedometer – based PA for the workdays and non-workdays was respectively 4260 ± 716, 3947 ± 414.

Fit indices and reasonable values of these indices (GFI=0.95, AGFI=0.91, CFI=0.9, RSMEA=0.07) showed this model was to fit data.

**Figure 1:**
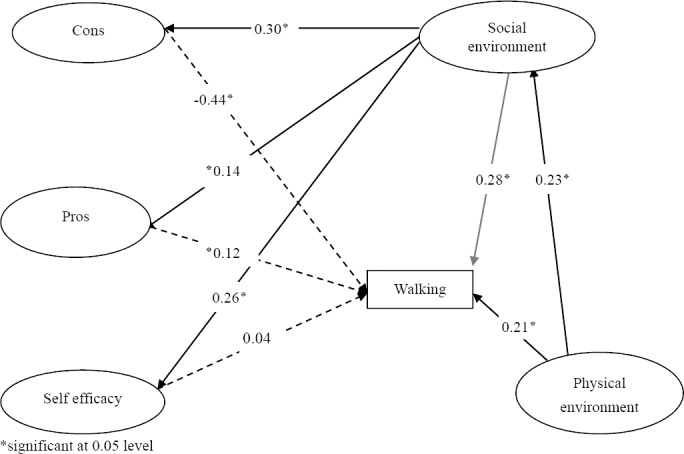
Psychological, environmental and social influences on walking. GFI=0.95, AGFI=0.91, RSMEA=0.07, CFI=0.9

The Results of environmental, social and Psychological effects on walking are displayed on Figure 1.

The Present study identified that psychological factors (self-efficacy, cons, Pros), physical environment and social environment affected walking directly, also social environment affected walking through cons, pros and self- efficacy indirectly and physical environment affected walking through social environment indirectly.

The path coefficients were shown in Figure 1. According to path coefficient, cons were the most predictor of walking behavior. Also physical and social environment were important factors that affected walking after the cons. All of the paths were significant except self-efficacy–walking.

**Table 2:** The total, direct and indirect effect of social, physical environment and personal factor on walking

	direct	indirect	total
Cons - Walking	-0.44*	-	-0.44
Pros - Walking	0.12*	-	0.12
self efficacy - Walking	0.04		0.04
Social environment-walking	0.28*	-0.12	0.16
Physical environment-walking	0.21*	-0.04	0.17

* significant at 0.05 level

### 3.1 Moderate Physical Activity Excluding Walking

**Figure 2:**
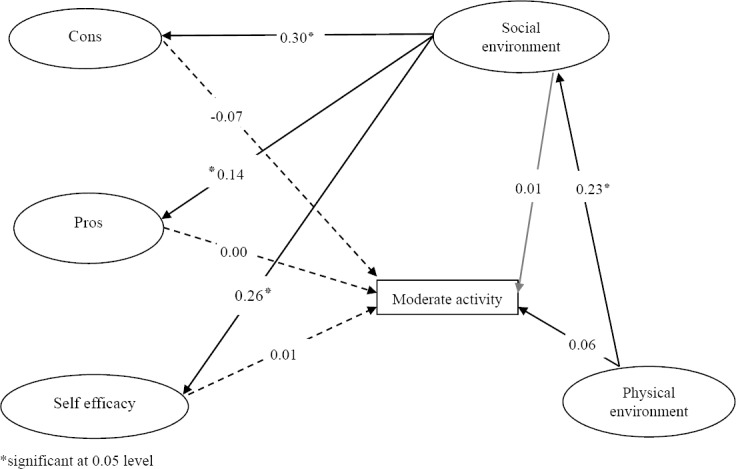
Psychological, environmental and social influences on moderate activity

Fit indices and reasonable values of these indices (GFI=0.90, AGFI=0.92, CFI=0.91, RMSEA=0.08) showed this model was to fit data. Figure 2 displays the results of physical, social environment, and psychological effects on moderate PA was similar to figure 1. There were not statistically significant differences between moderate activity and social, physical environment and psychological factors cons, unlike the results of walking.

The Present study identified psychological factors (self-efficacy, cons, Pros), Physical environment and social environment affected moderate activity excluding walking directly, also social environment affected moderate activity excluding walking indirectly through cons, pros and self-efficacy, and physical environment affected moderate activity excluding walking indirectly through social environment. The Path coefficients were shown in Figure 2.

**Table 3:** The total, direct and indirect effect of social, physical environment and personal factor on**** Moderate physical activity excluding walking

	Direct	Indirect	Total
Cons - Moderate activity	-0.07	-	-0.07
Pros - Moderate activity	0.00	-	0.00
Self efficacy - Moderate activity	0.01	-	0.01
Social environment- Moderate activity	0.01	0.06	0.07
Physical environment- Moderate activity	0.06	-0.01	0.05

* significant at 0.05 level

### 3.2 Vigorous Activity

**Figure 3:**
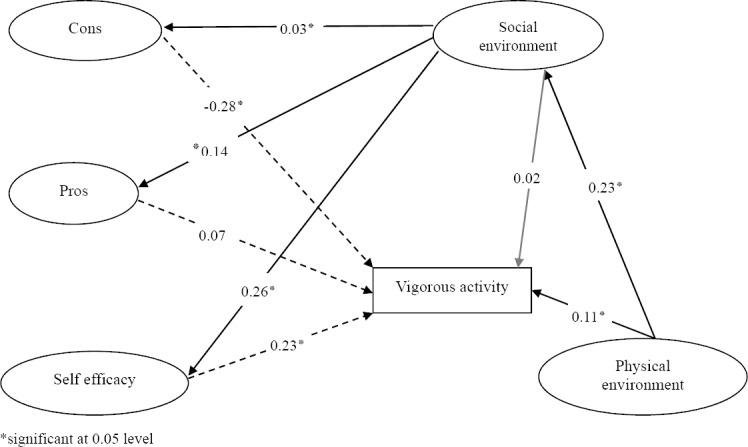
Psychological, environmental and social influences on vigorous activity

Fit indices and reasonable values of these indices (CFI=0.91, AGFI=0.90, GFI=0.95, RMSEA=0.08) demonstrated this model was to fit data. Figure 3 demonstrates the effects of abovementioned variables on vigorous activity. This model was similar to the walking and moderate activity excluding walking model. This study identified cons, self-efficacy and physical environment which were significantly related to vigorous activity. The Present study identified psychological factors (self-efficacy, cons, Pros), Physical environment and social environment affected vigorous activity directly, also social environment affected vigorous activity indirectly through cons, pros and self-efficacy, and physical environment affected vigorous activity indirectly through social environment. The Path coefficients were seen in figure 3. According to the path coefficient cons and self-efficacy were the most factors to predict of vigorous activity behavior.

**Table 4:** The total, direct and indirect effect of social, physical environment and personal factor on**** vigorous activity

	direct	indirect	total
cons-vigorous activity	*-0.28	-	-0.28
pros-vigorous activity	0.07	-	0.07
self efficacy-vigorous activity	*-0.23	-	0.23
social environment -vigorous activity	0.02	0.16	0.18
Physical environment-vigorous activity	*-0.11	0.04	0.15

* significant at 0.05 level

## 4. Discussion

Although most of the researches have focused on personal factors, social ecological models consider social and physical factors accompanied by personal variables to increase PA (King et al., 2002; Hall & McAuley, 2010). The present study applied path analysis to identify the appropriate strategy for improving PA; in this study cons were the most important factor to predict PA behavior which conforms to scientific base (Glanz & Rimer, 2008). Other studies showed that perceived barriers were important factors to decrease PA in women (Khan et al., 2002; Pan et al., 2009; Shibata et al., 2009). Social and physical environment were priority factors to predict PA after the cons. In this study, personal factors, social and physical environments affected all forms of PA directly, moreover social and physical environment indirectly affected PA through personal factors and social factor respectively. The study of McNeill et al. (2006) indicated physical environment affected PA not only directly, but also indirectly through social factors (consist with the present study) and personal factors. Other studies revealed PA has been influenced directly and indirectly by social and physical environments (Elder et al., 2007; Bolívar et al., 2010; Ogilvie et al., 2011). However Elder et al. (2007) considered direct and mediator role of social, physical environment and psychological factors to promote PA. It can be concluded that in social ecological models different multilevels affect each other. The present investigation indicated that physical environment had direct and indirect effects on walking, moderate and vigorous activity. The studies suggested availability of PA facilities, quality of a neighborhood (Duncan & Mummery, 2005; McNeill et al., 2006; CDC & Prevention, 2007; Cleland et al., 2010). Safety, ease of exercising (Duncan & Stoolmiller, 1993), attractive neighborhood (Frederick & Ryan, 1993; Shibata et al., 2009) are associated with walking. However, these results demonstrate physical environment has a positive role in PA promotion. In the current study social environment was significantly related to walking, also in previous studies (Sallis et al., 1992; Sallis et al., 2000; Shibata et al., 2009) peer support was an important factor on PA and Sallies et al. (2000) found that PA and peer support had indeterminate relationship. In the current study social environment both directly and indirectly affected PA through cons, pros and self-efficacy. This consisted of other studies that reported social support influenced PA trough self-efficacy (Rovniak et al., 2002; McAuley et al., 2003; McNeill et al., 2006; Motl et al., 2007) and motivation (Shibata et al., 2009; Ishii et al., 2010) as proposed in theoretical model, social support influenced both directly and indirectly PA. In accordance with the above-mentioned, it might be concluded that the utility of social ecological model could change researches to be multilevel and identify the influences of personal and ecological factors on behavior.

## 5. Study Limitations and Delimitations

The current study has limitations PA measure may be overlap between walking and moderate activity. Second this study examined some of the variables and omitted some variables (e.g. social norm). Third, this study was carried out based on a sample of women employees from Tabriz, Iran. This issue might reduce the generalizability of findings to other parts of Iran and other groups of women such as homemakers. The difficulty is further because the target group of this study was from age range of 24-55 and had a high level of education and on the other hand sample size was small, because the pedometer is expensive in this country.

The psychological, environmental and social factors based on social ecological models were used to understand PA behavior. “Such correlates could have been added to the model to better understand its relationship to activity in the presence of other factor” This study has several delimitations. Also we used structural equation model to test social ecological models on PA. Path analysis, the direct, indirect and total effects of factors on PA was done for the first time in Iran. The current study identifies that psychological, physical and social factors could be shown to have direct and indirect influences on all forms of activity. The barriers of PA were the most predictor of this behavior, and based on results, it can be concluded that decreasing the barriers along with improving social and physical environment can lead to increasing PA and health promotion.
